# Games of uncertainty: the participation of older patients with multimorbidity in care planning meetings – a qualitative study

**DOI:** 10.1186/s12877-021-02184-z

**Published:** 2021-04-13

**Authors:** Jannike Dyb Oksavik, Marit Solbjør, Ralf Kirchhoff, Maren Kristine Raknes Sogstad

**Affiliations:** 1 Department for Health Sciences, Faculty of Medicine and Health Sciences, Norwegian University of Science and Technology, Institutt for helsevitenskap, NTNU i Ålesund, Ålesund, Norway; 2 Department of Public Health and Nursing, Trondheim, Faculty of Medicine and Health Sciences, Norwegian University of Science and Technology, Institutt for samfunnsmedisin og sykepleie, NTNU, Øya Helsehus, Mauritz Hansens gate 2, Trondheim, Norway; 3 Department for Health Sciences, The Centre for Care Research, Faculty of Medicine and Health Sciences, Norwegian University of Science and Technology, NTNU i Gjøvik, Gjøvik, Norway

**Keywords:** Multimorbidity, Delivery of health care, integrated, Patient care planning, Patient participation, Game theory, Uncertainty, Goal-oriented care

## Abstract

**Background:**

Active patients lie at the heart of integrated care. Although interventions to increase the participation of older patients in care planning are being implemented in several countries, there is a lack of knowledge about the interactions involved and how they are experienced by older patients with multimorbidity. We explore this issue in the context of care-planning meetings within Norwegian municipal health services.

**Methods:**

This qualitative study drew on direct observations of ten care-planning meetings and an interview with each patient right after the meeting. Following a stepwise-deductive induction approach, the analysis began inductively and then considered the interactions through the lens of game theory.

**Results:**

The care-planning interactions were influenced by uncertainty about the course of the disease and how to plan service delivery. In terms derived from game theory, the imaginary and unpredictable player ‘Nature’ generated uncertainty in the ‘game’ of care planning. The ‘players’ assessed this uncertainty differently, leading to three patterns of game. 1) In the ‘game of chance’, patients viewed future events as random and uncontrollable; they felt outmatched by the opponent Nature and became passive in their decision-making. 2) In the ‘competitive game’, participants positioned themselves on two opposing sides, one side perceiving Nature as a significant threat and the other assigning it little importance. The two sides negotiated about how to accommodate uncertainty, and the level of patient participation varied. 3) In the ‘coordination game’, all participants were aligned, either in viewing themselves as teammates against Nature or in ascribing little importance to it. The level of patient participation was high.

**Conclusions:**

In care planning meetings, the level of patient participation may partly be associated with how the various actors appraise and respond to uncertainty. Dialogue on uncertainty in care-planning interventions could help to increase patient participation.

**Supplementary Information:**

The online version contains supplementary material available at 10.1186/s12877-021-02184-z.

## Introduction

Older patients with multimorbidity, suffering from two or more chronic diseases, often have complex health care needs [1–3]. This complexity means that goals for the services patients receive are not always unified among actors, nor do they always align with patients’ own preferences [1, 4, 5]. Furthermore, patients’ decision-making abilities tend to decline with age and the cumulative effects of long-term diseases which presents challenges for achieving patient participation in care planning [1, 6]. Nevertheless, person-centered, integrated care is the gold standard for service delivery, even when achieving it may be challenging [7, 8]. Integrated care is a structured effort to provide coordinated, pro-active, multidisciplinary, and person-centered care [2, 9, 10]. Person-centered care can be operationalized through goal-oriented care, in which health professionals and patients identify and discuss what matters most to patients and align the goals for care with patients’ preferences, values, and needs [8, 11, 12]. Patients’ goals may relate to reducing symptoms or improving physical functioning or well-being; they can also have social dimensions or reflect life values [5, 8]. Goal-oriented care planning is assumed to increase patients’ self-management abilities, health maintenance, and experience of care quality [1, 5, 11, 13].

However, the delivery of integrated care in general, and the achievement of patient participation in care-planning meetings in particular, is yet to be optimized. Older patients generally wish to participate more than they are allowed to do [14, 15]. Patients have reported a range of facilitators of and barriers to participation [16, 17]. The readiness of patients with multimorbidity to participate depends on, among other things, their physical and emotional strength and support from relatives [17, 18]. Patients may lack knowledge about goal setting, the rehabilitation process, and their condition; consequently, they can feel too disempowered to participate [4]. Patients have also reported difficulties in interacting with health professionals, including unsupportive attitudes regarding their beliefs and abilities related to care management, lack of information, and disagreements about the plan of care [17]. Health professionals and patients interpret and frame health problems differently [1]. What is more, health systems are changing to favor shorter hospital stays, with more services delivered in patients’ own homes [19]. Patient participation can be challenging, additionally, when care planning occurs early in a patients’ illness trajectory because some patients have less desire to participate when their conditions are acute and they have a higher number of diagnoses [4, 6]. Following acute illness, patients’ preferences may also change [20].

Patients’ experiences of multimorbidity are often characterized by a state of flux, in which self-management priorities can change from day to day [1, 3]. The suffering from multimorbidity can be greater than the sum of its parts; it is an encounter with complexity because illness impacts both bodily and emotional health and brings social consequences [3, 17]. For these patients, the future is uncertain because chronic disease can take different courses: most typical in old age is prolonged gradual decline in physical function from an already low baseline. Otherwise, illness trajectories can be punctuated by episodes of acute deterioration and some recovery [21]. Declining physical capacity in older individuals often manifests itself in falls and fall-related injuries [22]. There is a risk that minor physical events can be fatal for patients when they occur in combination with declining reserves [21]. Because the actors' perceptions of the situation may differ, achieving patient participation can be particularly challenging in this patient group. For health professionals to enable patients to participate in the care-planning process, they need to take individual capabilities, preferences, and perceptions of illness into consideration [2, 18].

To facilitate integrated care, more knowledge is needed about what is happening in care-planning conversations and how to overcome interactional difficulties to understand patients’ perspectives [1, 23, 24]. This study explores a care-planning intervention in Norwegian municipal health services, focusing on patient participation through two research questions:

What is the patients’ role in care-planning meetings?

How do patients experience participating in care-planning meetings?

## Methods

### Design

This qualitative study is inspired by constructivism, which explores the realities people construct and the implications of those constructions for individuals’ interactions with others [25]. To capture the interactions and experiences involved in patient participation, we combined direct observations [25] of ten care-planning meetings and individual interviews with the patients immediately after participation in the meeting. Observations are particularly suitable for exploring interactions [26] because they provide opportunities to describe the setting, activities, and actors in detail, thereby allowing a better understanding of the context [25]. Individual interviews provide insights into the patients’ experiences of these meetings. The analysis aimed for concept development through a process called stepwise-deductive induction [26].

### The Norwegian context and the care-planning intervention

In Norway, services for older people are broadly accessible and primarily financed, organized, and delivered by public entities in the municipalities [27]. This study includes health services in community hospitals, nursing homes, and patients’ homes. The care-planning intervention was carried out as follows: After an individual patient experiencing an acute episode of disease was allocated health care services by the municipality, the patient was invited to participate in planning how these services could be delivered. Health professionals asked, ‘What matters to you?’ as a basis for a conversation about what was important to each individual patient [28, 29]. The patient and health professionals formulated a goal to work towards over the following weeks. Care planning with patients occurred either in conversations with one health professional or during interprofessional meetings. The intervention could be repeated in later care-planning meetings.

### Recruitment and sample

We purposively chose four municipalities that had implemented the intervention. Two municipalities were urban areas with 40,000 and 70,000 inhabitants, respectively, while two rural municipalities had 2000–3000 inhabitants. We observed meetings in clinical settings occurring independently of the present study. We aimed for a purposive sample of meetings in different kinds of wards involving patients in different stages of illness trajectories. Managers at the wards asked the eligible patients to participate, and the patients were approached face to face. The inclusion criteria were patients having multimorbidity and newly emerged needs for health and care services so that care planning was needed. The age of the patients was determined by the municipalities’ routines; the intervention was applied primarily for patients over 80 years of age but could be used for younger patients in rehabilitation wards if they had complex needs. The exclusion criteria for the current study were cognitive impairment and short life expectancy. The number of patients who refused to participate in the study was not counted because we could not control whether the health professionals who recruited patients declined to invite certain patients. However, we did not aim for a representative sample.

### Data collection

From October 2018 to December 2019, the first author carried out direct observations of care-planning meetings and conducted patient interviews. During these observations, the researcher attempted to assume a neutral role and filled out an observation guide about the structure of the meetings and patient participation during the meetings (Additional file 1). The meetings lasted for 41 min on average. Observations were also carried out before and after the meetings, and informal talks with health professionals provided additional information about the context. Field notes were written after each observation and interview.

The interviews with patients were carried out in patient’s rooms or meeting rooms on the wards or in their homes. The semi-structured interview guide [30] focused on patients’ experiences of participation in care-planning meetings (Additional file 1). Neither patients’ relatives nor health professionals were present at the interviews, which lasted for 36 min on average. The length of each interview was adjusted to the energy level of the patient. One of the interviews was conducted by telephone. The interviews and meetings were audio-recorded and transcribed verbatim by the first author. The last meeting was not audio recorded, but thorough notes related to the observation guide and citations were written. After observation of the ten meetings, patterns were detected in how the meetings were organized and carried out across the different settings, and the material was considered substantial enough to convey information about the intervention.

### Analysis

Stepwise-deductive induction is based on grounded theory. In this process, the analysis begins inductively and subsequently draws on existing theory in concept development. We chose this method because it aims to elaborate new ideas from empirical data. Moreover, the analysis involved is more linear than in grounded theory [26]. The stages of the analysis are 1) empirical close coding, 2) grouping codes to subcategories, 3) merging subcategories with theory, and 4) concept development. The coding process is iterative between adjacent stages. Elements in the empirical data that trigger analytical ideas are recorded in memos [26].

The first author carried out the analysis in regular discussion with the co-authors. Firstly, she coded the transcripts by labeling small sections of text, resulting in 530 inductively based codes. The coding was more focused than that described by Tjora [26], as our codes were meant to convey meanings that could help to answer the research questions. The field notes were not coded but provided contextual understanding for the authors. Secondly, codes were sorted into groups based on the level of coherence in each group; see the example of coding in Table 1.

**Table 1:** Example of codes

Empirical close codes	Code group
‘Cannot let you know if I faint, it happens so fast’. Scared by the risks surrounding the symptoms. Wanted to live at home but did not manage to. Unsure how long things will continue to go well. I wish to await the decision about rehabilitation service at home. My symptoms determine the plans.	Patients’ experience of uncertainty
But you just came here; you may recover quickly. Your symptoms are common in old age. ‘We can predict that your situation will improve’. We test if the patient is ready to go home through a few days’ home visit. We will do anything for you to be safe at home.	Health professionals’ efforts to take control of uncertainty

All authors discussed the code groups and how to interpret the emerging patterns, as well as discussing the different roles of participants.

#### Inclusion of theory

Thirdly, code groups were linked to theory. In particular, game theory was deemed to be relevant because games can serve as a metaphor through which to understand patient participation [31, 32]. The more precise term for game theory is ‘interactive decision theory’ or ‘theory of interdependent decision making’ [33–35]. According to this theory, the encounter between patients and health professionals can be understood as a two-way interaction in which the outcome is affected by the actions and choices of each participant, leading to different types of games [32, 36, 37]. The roles of the players can be those of teammates, contenders, opponents, decision-makers, or subordinates [33, 38]. The interaction patterns in our data correspond to three kinds of games found in theory. The categories of these game types were developed by going back and forth between the empirical data and the theory.

During the following conceptualization, we chose to ‘zoom in’ [39] on the coding groups relating to uncertainty in decision-making. In game theory, ‘uncertainty’ means that the outcomes of decision-making do not depend solely on the actions of the players but rather are subject to the invisible hand of chance. This element of randomness can be depicted as resulting from the moves of an imaginary player: Nature [33]. We examined how the informants assessed uncertainty in care planning by looking for statements reflecting beliefs about whether and how one could plan care and the likelihood that these plans would come to fruition. Finally, we examined levels of patient participation. At a low level, patients sought or received information without participating in decision-making. At a medium level, the collaboration involved dialogue, but health professionals made the final decisions. A high level of participation involved shared decision-making based on patients’ preferences, medical evidence, and clinical judgment [4, 23, 36, 40]. Additional file 2 more thoroughly describes the conceptual framework. The data were managed using NVivo [41] software.

## Results

Ten patients participated. Their mean age was 88 years. Eight of the patients had been hospitalized during the current disease episode. The main health problem that had led to contact with health services was in each case intertwined with other diagnoses. All patients had functional decline, and none could walk without aids or help. Two of the patients had a salient mental diagnosis. Table 2 provides an overview of the study participants.

**Table 2:** Characteristics of the ten care-planning meetings and the patients

Patient’s gender, age	Patient’s main health problem, number of diagnoses, and ward	Participants in the care-planning meeting
P#1 Female, 86 years	Fractured arm. > 2 diagnoses. Start of stay at rehabilitation/intermediate care unit, city municipality.	Patient and a nurse.
P#2 Female, 96 years	Chest pains and abdominal pain. > 4 diagnoses. Start of stay at rehabilitation/intermediate care unit, city municipality.	Patient and a nurse.
P#3 Female, 97 years	Fall, fractured neck of femur, with infection. > 5 diagnoses. End of stay at rehabilitation ward, city municipality.	Patient, case manager from office handling allocation of services, physiotherapist, nurse, home care nurse, and daughter. Three nursing students observed the meeting.
P#4 Female, 98 years	Several falls assumed to be caused by orthostatic hypotension. > 2 diagnoses. End of stay at rehabilitation ward, city municipality.	Patient, case manager from office handling allocation of services, nurse at the ward, home care nurse, daughter, and adult granddaughter.
P#5 Female, 62 years	Pneumonia. > 5 diseases. Middle of stay at intermediate care unit, city municipality.	Patient, husband, case manager from office handling allocation of services, and nurse from the ward. One nursing student observed the meeting.
P#6 Female, 91 years	Weakened by cumulative effect of multiple conditions. > 5 diagnoses. Middle of stay at rehabilitation ward, city municipality.	Patient, case manager from office handling allocation of services, home care nurse, and ward nurse. Daughter and two sons.
P#7 Male, 94 years	Functional decline and emerging needs for home care services. > 5 diagnoses. Meeting at patient’s home before short-term stay at nursing home, rural municipality.	Patient, wife, and nurse in home care services.
P#8 Female, 96 years	Syncope. > 2 diagnoses. Meeting at patient’s home, right after stay in intermediate unit, rural municipality.	Patient and nurse in home care services.
P#9 Female, 86 years	Hip surgery. > 5 diagnoses. Middle of stay at short-term ward, rural municipality.	The patient did not wish to participate in the meeting. Four daughters, head nurse at care home, physiotherapist, and nurse.
P#10 Male, 75 years	Fractured neck of femur. > 4 diagnoses. End of stay at short-time ward, rural municipality.	Patient, head nurse in home care services, physician, physiotherapist, mental health nurse, case manager, daughter, son, and nurse from home care services.

### The care-planning games of uncertainty

The objective of the care-planning intervention in this context was to agree on a rehabilitation goal that will facilitate the patient’s discharge to his or her home. The care-planning meetings took place early in the recovery process, in most cases following a hospital admission and change in functional status. The meetings were mainly discussions to gain an overview of patients’ medical symptoms and practical problems related to declining functional abilities.

In the context of game theory, the patterns of interaction between the actors in these meetings can be understood as games with four kinds of players. The first was the patients, who had unsolved, inconclusive disease symptoms and required ongoing medical treatment. They often played under difficult conditions, being in an uncertain and confusing situation. Moreover, the interviews indicated that the patients felt disoriented about which services they would receive and when. These were decisions in which they perceived themselves to have little influence. The patients attempted to be cooperative players. Health professionals, the second kind of players, often drove the meetings, which began with each health professional presenting an evaluation of the health status of the patient. Health professionals played on their home ground: they had an overview of the situation and knew the rules of the game and the routines prescribed by the intervention. They were also the ones who pushed the decision-making to a conclusion. The third kind of player was relatives. Because the intervention does not specify questions addressed directly to relatives, relatives were assigned the role of observers who provided information and helped the patients. Sometimes they also acted as advocates for the patients, taking on a more active role.

The fourth player in the game is the imaginary player called **‘**Nature’, an objective force with the power to change the plans for service delivery when incidents such as disease, or improvements in health, occur by chance. Nature acts in unpredictable ways, leading to uncertainty. Although uncertainty is always present as a factor, the players assessed its importance differently, and these differences affected their planning of care. For example, if a patient had previously suffered a fall, the players considered whether to account for the possibility of further falls. The players’ different approaches to care planning shaped their arrangements, roles, and interactions. In particular, varying perceptions of the level of uncertainty and its importance in the game among players, and consequently how their actions related to Nature, shaped three different types of game: the game of chance, the competitive game, and the coordination game. The different games represent interaction patterns observed in the care-planning meetings. Different games could be played out in the same meeting, depending on the topic that were discussed. In the following, we describe the characteristics of each game.

#### The game of chance

In care-planning decisions that followed the pattern of the game of chance, patients seemed to perceive future events as uncontrollable and random. They felt outmatched by the opponent Nature, believing that the course of the disease and what happened within the health system would be dictated by chance. Their health professionals and relatives were relegated to the role of spectators on the sideline, in the sense that the outcome of care planning was understood to be determined more by Nature’s actions than by the patients’ own will or engagement in decision-making with other players. Consequently, when health professionals asked patients in this category what mattered to them, the patients were passive and expressed few preferences. They became receivers of information about the plans and goals that health professionals and relatives set for them.

In the interactions observed, several patients anticipated a deterioration of health, expressing fear of incomprehensible symptoms, pain, or severe illness. Many had already experienced sudden health deterioration in the form of falls or acute hospital stays:

Patient: I really hope the infection stays under control so that I can go through with this. This is the fifth time the operation has been scheduled. (meeting, P#5).

Moreover, patients felt unable to predict their level of physical strength or tiredness from day to day, meaning they did not know how active they could be in the recovery process. Patient 9, for example, suggested that her well-being was beyond her control:

Interviewer: So you were at the hospital not too long ago?

Patient: Hip surgery. And it went just fine. Now afterwards, it’s been a big mess. I fell a few times.

Interviewer: Oh, you have, huh? I see.

Patient: It was all going so well when I got back, but then things just took a turn. I don’t know what caused it. (interview, P#9).

This patient chose not to attend the care planning meeting. Other patients’ feelings of uncertainty appeared when they agreed only doubtfully to health professionals’ plans, making qualifying statements such as ‘I hope..’, ‘we will see if..’, ‘I’ll try’, and ‘if something does not occur’. Through the lens of game theory, these interactions appear as ones in which the role of Nature was understood to be strong, meaning that patients could not predict the outcomes of their available choices. Those patients who appeared to experience the greatest levels of uncertainty did not look forward or articulate any health-related goals:

Case manager: What do you think if you look ahead a bit, what is important to you in the situation you are in now?

Patient: Just that you all keep being good to me and, well, I don’t feel so positively about me getting better. (meeting, P#6).

Patients’ expressions of uncertainty, fear, or a sense of chaos were little explored or discussed by the health professionals, whose moves were, rather, to calm patients down and emphasize their own control of the situation:

Patient: It all just snowballs.

Nurse: And I think it’s important for you and [*spouse*], now that you are juggling a lot of things at once what with your ear and your stomach and your back that you had looked at a few days ago, that you try to focus on only one thing at a time. And right now, it’s the surgery. Have some fun this weekend.

Patient: Ok, ok.

Nurse: Come back on Monday. We have it under control. We will help you with what you need. And only focus on that. When that’s done with...we’ll take the next thing. If you think about everything it’ll just create this chaos in your ...

Patient: I know, I know. But I have to say, I’m dreading that operation, because she said so many things that could go wrong if...but that was only a percentage, of course. Even paralysis.... (meeting, P#5).

Health professionals emphasized areas in which the patients’ health was good and pointed out the activities the patient could manage in their daily life. They also offered security by placing safety alarms in the patients’ homes in case of critical events or asking patients about what they needed to feel safe. However, in the language of game theory, the safety offered by health professionals was insufficient to defeat the player Nature. The patients were subordinated to Nature and consequently to other players as well because of their passivity in decision-making.

#### The competitive game

In care-planning decisions that followed the pattern of the competitive game, the players formed two sides: one side perceived Nature as a significant threat, emphasizing the high degree of uncertainty in the care trajectory and worrying about how to plan for the risk of deteriorating health. The opposing side was less preoccupied with Nature. The courses of action proposed by each side differed, and the two sides consequently disagreed about the patients’ need for services.

Youngest son: Then I would like to take it a step further: if she isn’t functioning well enough to come home—then what do you do?

Coordinator: Then we apply for a different living situation. Right? Like a different level of care. Yeah. But we’re not there yet, no. *(light laughter).*

Youngest son: Right, no. But just to have asked that question in time.

Coordinator: Right. Well, we’ll deal with it when it’s ... (…).

Youngest son: Well, I still think it’s relevant to ask that. She is nearly 92, after all. (meeting, P#6).

These competitive games ended with winners and losers in decision-making because one of the sides disagreed with the final decisions. The level of patient participation depended on which side of the game the patient was on.

How the players distributed themselves between the two sides varied. Often, relatives wanted more health services for the patient, either because they perceived a high level of risk in the patient continuing to live at home or because they were exhausted by helping. The health professionals aligned themselves against these preferences when they did not accord with the routines and resources available. Another division of players could occur if the patient did not align himself against Nature when the other players all did. For example, the relatives could form an alliance with health professionals to persuade the patient to receive more health services in order to manage everyday life or reduce the risk of adverse events. Even when the patients seemed unaware of or untroubled by that risk, they had minimal opportunity to influence the decisions.

Nurse: Is there something you have been thinking about that might be important to you that you can tell us, something you’d like to continue with or achieve?

Patient: It’s a little difficult, that, right now.

Wife: I think it’s important for you, I have to say, that I am there to help you. (…) I’m the one responsible. You wouldn’t manage alone. (…).

Nurse: Have you given that any thought? *(short silence)* Is there something she does for you that we at home care services can help you with?

Patient: No, that would ... What might that be?

*(15 s silence)*

Nurse: You can’t think of anything? (meeting, P#7).

In cases such as these, the negotiation between opposing sides overshadowed the focus on the patients’ values and preferences in care planning.

#### The coordination game

In care-planning decisions that followed the pattern of the coordination game, all players either aligned themselves as teammates against Nature or else did not ascribe much importance to the forces represented by Nature. Patients, health professionals, and relatives coordinated their care-planning strategies to accommodate uncertainty and risk, thereby arriving at a shared goal for care. When the players assessed the risk of health deterioration to be high, viewing Nature as a strong opponent, they planned to stay on the safe side and collaboratively discussed fears and contingencies. The dialogue also elicited how each of the players perceived risk.

Grandchild: ... We’ll have to discuss it with the home care services, I think. Maybe get more frequent visits and ...

Daughter: But she is scared at home, you know.

Coordinator: It’s all the hours when you aren’t here, that’s a lot of hours in a day.

Home care nurse: And the nights, especially.

Coordinator: A day center is an alternative, but that still won’t cover all 24 h, you know. It’s about finding a solution. Yes.

Grandchild: I’m sure there is. There is always a solution.

Coordinator: It’s just that ...you feel unsafe being at home.

Patient: Yes, and I never know what might happen. (meeting, P#4).

This dialogue ended with agreement among the players that long-term care in a nursing home was the best solution. The patient repeated in the interview that she preferred this option.

In an opposite sort of scenario, the game could unfold as if Nature were not present; all players perceived the level of uncertainty to be low, and they assessed the situation as uncomplicated. This version of the game may have occurred because patients’ diseases were less complex, as in the case of a woman with a broken arm.

Coordination games were characterized by the time taken to share perceptions of uncertainty and to talk about the available options for care. The players did not form factions through the decision-making process, and they agreed on goals. The patients themselves were active and equivalent to other players in the decision-making.

### The concept of the game of uncertainty

Table 3 sums up the characteristics of the different types of games that unfolded depending on how the players assessed uncertainty. We suggest that perceptions of uncertainty were associated with different patient roles and levels of patient participation. This observation forms the point of departure for our discussion.

**Table 3:** The concept of the game of uncertainty

	Game of chance	Competitive game	Collaborative game
**Uncertainty**	The patient assessed uncertainty to a greater degree than other players. Temporal focus: did not look forward.	The players assessed the level of uncertainty differently.	The patient’s understanding of uncertainty was shared with other players. Temporal focus: the future.
**Participants’ roles**	Patient fighting alone against Nature.	Two sides, in which one of the sides saw Nature as an opponent.	All players were teammates, either aligning against Nature or not feeling threatened by it.
**Level of patient participation**	Low: the patient received information, was less active. Health professionals set goals for care.	Varied: depended on which side of the game the patient took. Difficult to agree on goals for care.	Higher: the patient functioned as an equal player within the team. Easier to agree on goals for care.

## Discussion

Goal-oriented care-planning interventions have been implemented to increase patient participation so that service delivery better aligns with patients’ values, preferences, and needs [8, 11, 12]. In some Norwegian municipalities, care planning is based on the question ‘What matters to you?’ [28, 29]. The present study explores the experience of participation for older patients with multimorbidity and the types of interactions involved. Decision-making interactions were shaped by different responses among players to the elements of uncertainty in the situation: the unknown course of the disease, unfamiliarity with the service delivery process, and the uncertain future self-management abilities of patients. Differences in how participants in the care-planning meetings assessed uncertainty, and thereby contended with the imaginary player Nature, led to the appearance in the care planning meetings of three different game patterns: the game of chance, the competitive game, and the coordination game. The level of patient participation was low in the game of chance, varied in the competitive game, and high in the coordination game. How each of the players accommodated uncertainty seemed to influence the patients’ opportunities and motivation to participate actively in care planning.

For the patients in the present study, uncertainty was central in the decision-making process and strongly affected the structure of the care-planning game. Previous studies investigating uncertainty in the context of patient participation have examined medical decision-making about prognosis and treatment options, mostly in patient–physician consultations [1, 42]. The influence of uncertainty has also been studied within the context of life-limiting chronic disease and cancer [43, 44]. A review of how integrated services for older people living at home address patients’ safety shows that safety is protected by preventing (unnecessary) health decline, polypharmacy, and uncoordinated service delivery [45]. However, although health and social care providers in thirteen case studies of European care programs thought they had sufficiently addressed safety issues, older people often still felt insecure [46].

According to game theory, assessments of uncertainty involve a feeling of ignorance about the future, meaning that the player cannot assign meaningful probabilities to the outcome of the game [33]. Similar descriptions can be found in previous health research suggesting that uncertainty about illness can affect patients’ temporal focus for a period: some patients focus only on current events and ignore the future. What is more, ignorance about the future reduces patients’ engagement and desire for information [43]. This issue needs attention from health professionals involved in care planning because the literature identifies patients’ perceptions of control as an internal factor important for their self-management in cases of chronic disease [24, 47].

The different game structures we have identified illustrate some underlying dynamics governing the interactions involved in care planning [32]. Game theory offers the advantage, in the present study, of illuminating how the players’ roles as passive participants, opponents, or teammates influenced the levels of patient participation in the three different types of game we observed. The first type, the game of chance, has a structure in which one player awaits the moves of another, more powerful player, Nature [33]. In the meetings we observed, patients who felt overwhelmed by Nature’s potential influence on their situations received information passively from health professionals, resulting in a low level of participation. Charles et al. [36] point out that many patients faced with serious illness, uncertainty about the outcome, and time pressure to make treatment decisions can feel extreme psychological and/or physiological vulnerability, which may make it difficult for them to participate in decision-making, no matter how well informed they might be [36]. This situation may apply to older patients with multimorbidity, in which illness is an encounter with complexity, affecting several areas of life and with an unknown course [3, 17, 21]. Feeling outmatched by the imaginary player Nature may influence the possibility of patients participating in their care planning and their motivation to do so.

The competitive version of the game had the structure of a competition between sides. This result is in line with studies showing that mismatches in the way patients and health professionals interpret and frame a patient’s health problems hamper patient participation [1]. One possible explanation for the occurrence of a competitive game is that this care-planning intervention focuses on self-management and health maintenance. In some cases, this focus excluded any dialogue about how the participants assessed age-related health deterioration and risks. The most typical illness trajectory in old age is prolonged gradual decline, often punctuated by episodes of acute deterioration and some recovery [21]. This raises questions about the feasibility of the intervention in the context of integrated care for older patients with multimorbidity. More work remains to be done on how to apply this intervention with this patient group to reach high-quality care through active participation.

Finally, coordination games have a structure in which the players coordinate their strategies [33]. This version of the game was the only one reaching the level of ‘shared decision-making’ [36, 40]; when uncertainty was high, all players related to it. Dialogue and an evaluation of options is an important component of shared decision-making [36, 43], and these elements allowed patients playing this game to participate despite the destabilizing presence of Nature. This dynamic was seen when some patients received support from health professionals through the segment of the meeting allotted to dialogue, which elicited differences in perceptions of uncertainty among the participants.

According to the ideals of integrated care, patients should be at the center of decisions about health service delivery, leading to greater self-management [2, 10]. This ideal may be difficult to achieve for some older patients with multimorbidity, as the acute phases of their diseases may be similar to those of patients with advanced illnesses in terms of how they deal with uncertainty during the course of the illness and in the future [43]. In the present study, the extent to which health professionals explored how patients assessed and handled uncertainty, thereby helping them to a better understanding of their situation, was low. For patients to become active participants—as the intervention requires—our study suggests that patients first need an overview of their own situation; it also illustrates the important role of health professionals in this process. Based on these arguments, the present study suggests that patient participation may increase if patient uncertainty is attended to in care planning.

### Strengths and limitations

The method of direct observation is influenced by the researcher’s perceptions and interpretations [25, 26]. Hence, the significance assigned in our analysis to uncertainty is one among multiple possible interpretations. It is known that in studies of patient participation researchers make attributions about the participants’ internal decision-making processes based on what the researchers observe [36]; this could be a limitation of the analysis conducted here. However, the triangulation of observations and interviews contributes to the credibility of the findings [25].

In the recruitment process, health professionals could have excluded patients with whom they perceived collaboration to be difficult. In addition, the observer could have influenced the meetings [25] if her presence led participants to behave more agreeably. Our study sample is too small to determine how often uncertainty appears in decision-making with this category of patients or the extent to which it influences patient participation. Other patterns of interaction may exist in such meetings which we were unable to capture with a sample of this size. Our concept is thus modifiable; we cannot draw conclusions as to whether a relationship exists between uncertainty, roles, and patient participation. However, our results are transferable to similar contexts insofar as they illustrate how interactions between elderly patients with multimorbidity and health professionals can be interpreted as a game in which uncertainty plays a part.

### Implications

Interventions aimed at facilitating patient participation do not automatically obtain their goal [14]. Patients’ individual beliefs and their perceptions of personal control influence decision-making and self-management [47]. To enable patients to participate, it may be beneficial to include a dialogue that elicits how uncertainty is assessed by the various participants in care-planning meetings. There are several specific ways in which this issue could be addressed in the intervention. First, questions could be included about whether and how patients perceive uncertainty within their situation. We found that health professionals used most of the time in the meetings to collect and share medical information. However, a different distribution of time in the meetings, with more time allocated to discussions of perceptions of uncertainty, might benefit some patients with complex needs. Second, decision-making and goal setting should be adapted to the patients’ temporal focus, that is, whether their focus is on the present or future [43]*.* Health professionals and patients can agree on the timeframe (e.g., days or weeks) for the plans they make. Making this dialogue an explicit component of care-planning interventions may increase person-centeredness and promote the alignment of service delivery with patients’ own goals. Keeping the game metaphor and the imaginary player Nature in mind may increase health professionals and patients’ understanding of care-planning interactions.

Because the influence of uncertainty does not apply to all patients equally, future research on the prevalence of this phenomenon is warranted. According to constructivist inquiries, concepts that are developed are open to continuous reconstruction because input from others leads to new or added meanings [48]. Further studies could refine the concept of uncertainty in care planning.

## Conclusions

The present study explores the experience of patient participation for older patients with multimorbidity in care-planning meetings within municipal health services. In the interactions observed, the actors’ assessments of uncertainty were salient in decision-making, and three patterns emerged, which we describe here, drawing on game theory, as three versions of the interaction ‘game’: a game of chance, a competitive game, and a coordination game. These interactions help us understand why some patients participate less in care planning than others. We conclude that care-planning interventions for older patients with multimorbidity should mandate that health professionals elicit and discuss uncertainty to achieve goal-oriented care based on patients’ preferences, values, and needs. Further research could explore the role of uncertainty in these meetings and how health professionals and patients can accommodate it in care planning.

## Supplementary Information

**Additional file 1.** Observation and interview guides.

**Additional file 2.** The emerging conceptual framework - Patient participation in the care-planning game

## Data Availability

The data generated and analysed in the current study are not publicly available due to Norwegian privacy legislation and the form signed by the participants about the study’s privacy.
