# Supplementary material for: Games of uncertainty: the participation of older patients with multimorbidity in care planning meetings – a qualitative study
Source: BMC Geriatr. 2021 Apr 13;21:242. doi: 10.1186/s12877-021-02184-z (PMC8045290; doi:10.1186/s12877-021-02184-z)
Supplement: Supplementary file 1 — Additional file 1. Observation and interview guides. [file 12877_2021_2184_MOESM1_ESM.docx]

**Additional file 1: Observation and interview guides.**

Observation guide for care planning meetings

This observation guide was filled out during and right after the meetings. However, the analysis of the meetings was mostly based on the transcripts of audio-recorded meetings.

| **THE MEETING** |  | **Interpretations of the researcher** |
| --- | --- | --- |
| Time | Time. Duration of the meeting |  |
| Description of the place | Where, how the place looked, what happened in the context. |  |
| Participants | Number of persons, their roles  Patient:  Health professionals:  Relatives: |  |
| Leader of the meeting |  |  |
| Structure of the meeting | How they carried out the meeting |  |
| Agenda | The agenda for the meeting   - According to written documents/tools - According to what health professionals and/or patients said during the meeting |  |
| Division of tasks |  |  |
| Did health professionals use tools or checklists? | Description of the tools and how they were used during the meeting |  |
| **Interactions** |  |  |
| Did participants know each other? |  |  |
| Atmosphere |  |  |
| Formal/informal conversation | Areas of the patients’ life and health they focused on |  |
| Communication | Professional terminology used?  Interruptions?  Room for asking questions?  Nonverbal communication |  |
| **What matters to you?** | Who asked the question?  What happened? |  |
| What health professionals did to let the patient participate |  |  |
| What the patient did to participate |  |  |
| Were there signs of less patient participation? |  |  |
| Assessment of how the researcher influenced the situation | *In most of the meetings, the researcher asked participants about their experience of being observed.* |  |
| Aspects that seem unclear and will be further examined (e.g., ask health professionals or patients after the meeting) |  |  |
| Emerging aspects in the meeting? |  |  |

Guide for interviews with patients

Can you please tell me a little bit about your previous experiences with the health services? Can you tell me about what happened when you got ill this time?

What matters to you regarding the services you are going to receive from now on? (Explore what kind of goals the patient has)

I was present at the meeting, but I would like to hear how you experienced the meeting?

What was the aim of the meeting?

Did you get to know ahead of the meeting what the purpose of the meeting was? Have you received information about which services there are for older people in the municipality? Is the information given by health professionals and their choice of words understandable?

Who in the meeting decided what you are going to receive help for? Why?

Were you allowed to say what matters to you? Do health professionals include your wishes in decisions about service delivery?

Do you participate in making decisions in your own care pathway? Do you wish to participate?

Was there anything else you wished to talk about in the meeting that you did not discuss?

Follow-up questions about what I observed in the meeting (e.g., asking for their interpretations of what happened and why).
