# Supplementary material for: Games of uncertainty: the participation of older patients with multimorbidity in care planning meetings – a qualitative study
Source: BMC Geriatr. 2021 Apr 13;21:242. doi: 10.1186/s12877-021-02184-z (PMC8045290; doi:10.1186/s12877-021-02184-z)
Supplement: Supplementary file 2 — Additional file 2. The emerging conceptual framework - Patient participation in the care-planning game [file 12877_2021_2184_MOESM2_ESM.docx]

Additional file 2: The emerging conceptual framework - Patient participation in the care-planning game

We use the metaphor of the game as a tool to study patient participation. What is often termed ‘game theory’ is more precisely referred to as ‘interactive decision theory’ or ‘theory of interdependent decision making’; it is not connected to ordinary games (Colman, 1995; Swedberg, 2001). Theories of patient participation and game theory both explain interactions and decision-making, but there are few studies of patient participation inspired by game theory. However, the use of game structures can provide valuable insights into the underlying dynamics of the interactions being studied – whether they be collaborative, conflictual, or threatening – and different actors’ functions and power in relation to one another (Tarrant et al, 2004; Colman, 1995). In conjunction with the theory of patient participation, the metaphor of games can help in understanding why some patients participate while others do not, illuminating obstacles to shared decision-making, which is the ideal of integrated, person-centered care (Leijten et al, 2018; Amelung et al, 2017; World Health Organization, 2016).

Patient participation

According to patient participation theory, goal-setting interactions between health professionals and patients should contain the following four elements to qualify as shared decision-making: 1) at least two participants are involved; 2) both parties share information that is relevant to the process, purposes, outcomes, and goals of care; 3) the patient discloses preferences, and both parties ask questions and evaluate options; and 4) they agree on the goals (Bunn et al, 2018; Rose et al, 2017; Charles et al, 1997). To put it another way, patient participation can be divided into three levels: low, medium, and high. This is shown in Table 1.

**Table 1: Levels of patient participation**

| Low | ‘Information-seeking/receptive’ |
| --- | --- |
| Medium | ‘Dialogue’ |
| High | ‘Shared decision-making’ is based on patients’ preferences, medical evidence, and clinical judgment. The parties agree on the decisions. |

(These levels are based on Rose et al, 2017; Kvæl et al, 2018; Charles et al, 1997 and Thompson, 2007).

For health professionals to enable patients to participate in the care process, they need to take individual capabilities, preferences, and perceptions of illness into consideration (Leijten et al, 2018; Vahdat et al, 2014). The encounter between patients and health professionals is a two-way interaction in which the outcome is affected by the actions and choices of each participant (Tarrant et al, 2004; Charles et al, 1997).

Care planning as a game

The game can be used as a metaphor to study decision-making (Goffman, 1961; Swedberg, 2001). This has been done in a few studies of health services, in which the decisions made in care pathways have been compared with a game in which the dynamics of the participants’ roles and the system made the management of care difficult (Allen et al, 2004). Here, Goffman (1961) describes games as social situations with some specific characteristics. The common elements of games include a purpose, procedures for action, rules, conventions regarding the number and roles of players, typical patterns of interaction, and results (Stenros, 2017). The present study focuses on the *roles* of players and their *patterns of interaction*.

The roles of the players can be those of teammates, contenders, opponents, decision-makers, or subordinates (Colman, 1995; Stenros, 2017). Unlike in classical game theory, players in real-world interactions are not simply rational actors; their motivations can have causes other than self-interest. Therefore, to understand a game, it is essential to explicate the beliefs, ideas, and experiences of the actors themselves (Swedberg, 2001; 48).

The patterns of interaction we found in our data correspond to three kinds of games. First, the game of chance, characterized by a pattern of interaction in which the outcomes are perceived to depend not solely on the decision-making of the players but also on an invisible hand of chance. This element of randomness can be personified in the moves of an imaginary player: Nature. Some literature has compared personal illness with the moves that Nature can make (Colman, 1995). In games of chance involving uncertainty, players do not know for certain the outcomes of the available choices and cannot even assign meaningful probabilities to them (Colman, 1995). The second game type is the competitive game, in which players’ preferences are mutually opposed. Goffman describes players’ interests as *sides*, an alignment that is unembodied and that is a function only of the game (Goffman, 1961). In these games, one player wins and the other loses (Colman, 1995). The third game type is the coordination game, in which the players’ preferences coincide; the players’ objective is to coordinate their strategies to obtain an outcome they all favor (Colman, 1995). We assume that how players interact to make decisions in different games can illuminate patterns of patient participation in care planning.

## References

Allen D, Griffiths L, Lyne P. Understanding complex trajectories in health and social care provision. Sociol Health Ill. 2004 Nov;26(7):1008-30. doi:10.1111/j.0141-9889.2004.00426.x

Amelung V, Stein V, Goodwin N, Balicer R, Nolte E, Suter E, editors. Handbook integrated care. Basel, Switzerland: Springer; 2017. doi:10.1007/978-3-319-56103-5

Bunn F, Goodman C, Russell B, Wilson P, Manthorpe J, Rait G, Hodkinson I, Durland M-A. Supporting shared decision making for older people with multiple health and social care needs: a realist synthesis. BMC Geriatr. 2018;18:165. doi:1186/s12877-018-0853-9

Charles C, Gafni A, Whelan T. Shared decision-making in the medical encounter: what does it mean? (or it takes at least two to tango). Soc Sci Med. 1997 Mar;44(5):681-92. doi: 10.1016/s0277-9536(96)00221-3.

Colman AM. Game theory & its applications in the social and biological sciences. Oxford: Butterworth-Heinemann Ltd.; 1995.

Goffman E. Encounters: two studies in the sociology of interaction. Bobbs-Merrill, Indianapolis;1961.

Kvæl LAH, Debesay J, Langaas A, Bye A, Bergland A. A concept analysis of patient participation in intermediate care. Pat Educ Couns. 2018;101(8):1337-1350. doi:10.1016/j.pec.2018.03.005

Leijten FRM, Struckmann V, van Ginneken E, Czypionka T, Kraus M, Reiss M, Tsiachristas A, Boland M, de Bont A, Bal R, Busse R, Rutten-van Molken M, on behalf of the SELFIE consortium. The SELFIE framework for integrated care for multi-morbidity: development and description. Health Policy. 2018;22(1):12-22

Rose A, Rosewilliam S, Soundy A. Shared decision making within goal setting in rehabilitation settings: a systematic review. Pat Educ Couns. 2017;100(1):65-75. doi:10.1016/j.pec.2016.07.030.

Stenros J. The game definition game: a review. Games Cult. 2017;12(6):499-520.

Swedberg R. Sociology and game theory: contemporary and historical perspectives. Theor Soc. 2001;30:301-35.

Tarrant C, Stokes T, Colman AM. Models of the medical consultation: opportunities and limitations of a game theory perspective. Qual Saf Health Care 2004;13:461-466. doi: 10.1136/qshc.2003.008417.

Thompson AGH. The meaning of patient involvement and participation: a taxonomy. Maidenhead: Open University Press; 2007.

Vahdat S, Hamzehgardeshi L, Hessam S, Hamzehgardeshi Z. Patient involvement in health care decision making: a review. Iran Red Crescent Med J. 2014 Jan;16(1):e12454. doi: 10.5812/ircmj.12454.

World Health Organization. Framework on integrated, people-centred health services. Report by the secretariat; 2016. Internet, retrieved 2020.12.28, available from: <https://apps.who.int/gb/ebwha/pdf_files/WHA69/A69_39-en.pdf?ua=1&ua=1>
